# Theories of Error Back-Propagation in the Brain

**DOI:** 10.1016/j.tics.2018.12.005

**Published:** 2019-03

**Authors:** James C.R. Whittington, Rafal Bogacz

**Affiliations:** 1 MRC Brain Network Dynamics Unit, Nuffield Department of Clinical Neurosciences, University of Oxford, Oxford OX3 9DU, UK; 2 Wellcome Centre for Integrative Neuroimaging, Centre for Functional Magnetic Resonance Imaging of the Brain, University of Oxford, Oxford OX3 9DU, UK

**Keywords:** deep learning, neural networks, predictive coding, synaptic plasticity

## Abstract

This review article summarises recently proposed theories on how neural circuits in the brain could approximate the error back-propagation algorithm used by artificial neural networks. Computational models implementing these theories achieve learning as efficient as artificial neural networks, but they use simple synaptic plasticity rules based on activity of presynaptic and postsynaptic neurons. The models have similarities, such as including both feedforward and feedback connections, allowing information about error to propagate throughout the network. Furthermore, they incorporate experimental evidence on neural connectivity, responses, and plasticity. These models provide insights on how brain networks might be organised such that modification of synaptic weights on multiple levels of cortical hierarchy leads to improved performance on tasks.

## Highlights

The error back-propagation algorithm can be approximated in networks of neurons, in which plasticity only depends on the activity of presynaptic and postsynaptic neurons.

These biologically plausible deep learning models include both feedforward and feedback connections, allowing the errors made by the network to propagate through the layers.

The learning rules in different biologically plausible models can be implemented with different types of spike-time-dependent plasticity.

The dynamics and plasticity of the models can be described within a common framework of energy minimisation.

## Deep Learning and Neuroscience

In the past few years, computer programs using **deep learning** (see Glossary) have achieved impressive results in complex cognitive tasks that were previously only in the reach of humans. These tasks include processing of natural images and language [1], or playing arcade and board games 2, 3. Since these recent deep learning applications use extended versions of classic **artificial neural networks** [4], their success has inspired studies comparing information processing in artificial neural networks and the brain. It has been demonstrated that when artificial neural networks learn to perform tasks such as image classification or navigation, the neurons in their layers develop representations similar to those seen in brain areas involved in these tasks, such as receptive fields across the visual hierarchy or grid cells in the entorhinal cortex 5, 6, 7. This suggests that the brain may use analogous algorithms. Furthermore, thanks to current computational advances, artificial neural networks can now provide useful insights on how complex cognitive functions are achieved in the brain [8].

A key question that remains open is how the brain could implement the **error back-propagation** algorithm used in artificial neural networks. This algorithm describes how the weights of synaptic connections should be modified during learning, and its attractiveness, in part, comes from prescribing weight changes that reduce errors made by the network, according to a theoretical analysis. Although artificial neural networks were originally inspired by the brain, the modification of their synaptic connections, or weights, during learning appears biologically unrealistic 9, 10. Nevertheless, recent models have demonstrated that learning as efficient as in artificial neural networks can be achieved in distributed networks of neurons using only simple plasticity rules 11, 12, 13, 14. These theoretic studies are important because they overrule the dogma, generally accepted for the past 30 years, that the error back-propagation algorithm is too complicated for the brain to implement 9, 10. Before discussing this new generation of models in detail, we first provide a brief overview of how the back-propagation algorithm is used to train artificial neural networks and discuss why it was considered biologically implausible.

## Artificial Neural Networks and Error Back-Propagation

To effectively learn from feedback, the synaptic connections often need to be appropriately adjusted in multiple hierarchical areas simultaneously. For example, when a child learns to name letters, the incorrect pronunciation may be a combined result of incorrect synaptic connections in speech, associative, and visual areas. When a multi-layer artificial neural network makes an error, the error back-propagation algorithm appropriately assigns credit to individual synapses throughout all levels of hierarchy and prescribes which synapses need to be modified and by how much.

How is the back-propagation algorithm used to train artificial neural networks? The algorithm is trained on a set of examples, each consisting of an **input pattern** and a **target pattern**. For each such pair, the network first generates its prediction based on the input pattern and then the synaptic weights are modified to minimise the difference between the target and the **predicted pattern**. To determine the appropriate modification, an error term is computed for each neuron throughout the network. This describes how the activity of the neuron should change to reduce the discrepancy between the predicted and target pattern (Box 1). Each weight is modified by an amount determined by the product between the activity of the neuron it projects from and the error term of the neuron it projects to.

Box 1 **Artificial Neural Networks**

A conventional artificial neural network consists of layers of neurons, with each neuron within a layer receiving a weighted input from the neurons in the previous layer (Figure IA). The input layer is first set to be the input pattern and then a prediction is made by propagating the activity through the layers, according to **Equation 1.1**, where ***x***_*l*_ is a vector denoting neurons in layer *l* and ***W***_*l *− 1_ is a matrix of synaptic weights from layer *l* − 1 to layer *l*. An activation function *f* is applied to each neuron to allow for nonlinear computations.

During learning, the synaptic connections are modified to minimise a cost function quantifying the discrepancy between the predicted and target patterns (typically defined as in **Equation 1.2**). In particular, the weights are modified in the direction of steepest decrease (or gradient) of the cost function (Figure ID). Such modification is described in **Equation 1.3**, where ***δ***_*l*+1_ is a vector of error terms associated with neurons ***x***_*l*+1_. The error terms for the last layer *L* are defined in **Equation 1.4** as the difference between the target activity ***t*** and the predicted activity. Thus, the error of an output neuron is positive if its target activity is higher than the predicted activity. For the earlier layers, the errors are computed according to **Equation 1.5** as a sum of the errors of neurons in the layer above weighted by the strengths of their connections (and further scaled by the derivative of the activation function; in **Equation 1.5** · denotes element-wise multiplication). For example, an error of a hidden unit is positive if it sends excitatory projections to output units with high error terms, so increasing the activity of such a hidden neuron would reduce the error on the output. Once the errors are computed, each weight is changed according to **Equation 1.3** in proportion to the product of the error term associated with a postsynaptic neuron and the activity of a presynaptic neuron.

Alt-text: Box 1

**Figure I:**
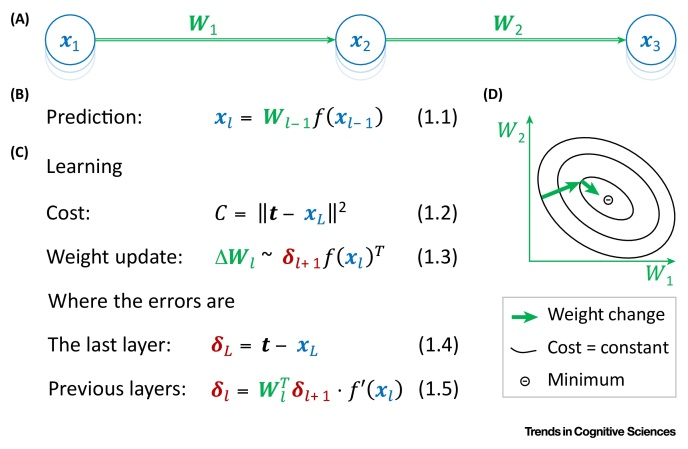
Artificial Neural Networks. (A) Layers of neuron-like nodes are represented by sets of stacked blue circles. Feedforward connections are indicated by green arrows. (B) Prediction. (C) Learning. (D) Schematic of the directions of two consecutive weight modifications (thick arrows) in the space of weights (for simplicity, only two dimensions are shown). Contours show points in weight space with equal cost function values.

Although the described procedure is used to train artificial neural networks, analogous steps may take place during learning in the brain. For example, in the case of the child naming letters mentioned above, the input pattern corresponds to an image of a letter. After seeing an image, the child makes a guess at the name (predicted pattern) via a neural network between visual and speech areas. On supervision by his or her parent of the correct pronunciation (target pattern), synaptic weights along the processing stream are modified so that it is more likely that the correct sound will be produced when seeing that image again.

## Biologically Questionable Aspects of the Back-Propagation Algorithm

Although the algorithmic process described above appears simple enough, there are a few problems with implementing it in biology. Below, we briefly discuss three key issues.

### Lack of Local Error Representation

Conventional artificial neural networks are only defined to compute information in a forward direction, with the back-propagating errors computed separately by an external algorithm. Without local error representation, each synaptic weight update depends on the activity and computations of all downstream neurons. Since biological synapses change their connection strength based solely on local signals (e.g., the activity of the neurons they connect), it appears unclear how the synaptic plasticity afforded by the back-propagation algorithm could be achieved in the brain. Historically, this is a major criticism; thus it is a main focus of our review article.

### Symmetry of Forwards and Backwards Weights

In artificial neural networks, the errors are back-propagated using the same weights as those when propagating information forward during prediction. This weight symmetry suggests that identical connections should exist in both directions between connected neurons. Although bidirectional connections are significantly more common in cortical networks than expected by chance, they are not always present [15]. Furthermore, even if bidirectional connections were always present, the backwards and forwards weights would still have to correctly align themselves.

### Unrealistic Models of Neurons

Artificial neural networks use artificial neurons that send a continuous output (corresponding to a firing rate of biological neurons), whereas real neurons use spikes. Generalising the back-propagation algorithm to neurons using discrete spikes is not trivial, because it is unclear how to compute the derivate term found in the back-propagation algorithm (Box 1). Away from the back-propagation algorithm, the description of computations inside neurons in artificial neural networks is also simplified as a linear summation of inputs.

## Models of Biological Back-Propagation

Each of the above-mentioned issues has been investigated by multiple studies. The lack of local error representation has been addressed by early theories by proposing that the errors associated with individual neurons are not computed, but instead the synaptic plasticity is driven by a global error signal carried by neuromodulators 16, 17, 18, 19. However, it has been demonstrated that learning in such models is slow and does not scale with network size [20]. More promisingly, in the past few years, several models have been proposed that do represent errors locally and thus more closely approximate the back-propagation algorithm. These models perform similarly to artificial neural networks on standard benchmark tasks (e.g., handwritten digit classification) 12, 13, 14, 21, 22, and we summarise several of them in more detail in the following sections.

The criticism of weight symmetry has been addressed by demonstrating that even if the errors in artificial neural networks are back-propagated by random connections, good performance in classification tasks can still be achieved 21, 23, 24, 25, 26, 27. This being said, there is still some concern regarding this issue [28]. With regard to the biological realism of neurons, it has been shown that the back-propagation algorithm can be generalised to neurons producing spikes [29] and that problems with calculating derivatives using spikes can be overcome [23]. Furthermore, it has been proposed that when more biologically realistic neurons are considered, they themselves may approximate a small artificial neural network in their dendritic structures [30].

There is a diversity of ideas on how the back-propagation algorithm may be approximated in the brain 31, 32, 33, 34, 35, 36; however, we review the principles behind a set of related models 11, 13, 14, 37 that have substantial connections with biological data while closely paralleling the back-propagation algorithm. These models operate with minimal external control, as they can compute the errors associated with individual neurons through the dynamics of the networks. Thus, synaptic weight modifications depend only on the activity of presynaptic and postsynaptic neurons. Furthermore, these models incorporate important features of brain biology, such as **spike time-dependent plasticity**, patterns of neural activity during learning, and properties of **pyramidal neurons** and cortical microcircuits. We emphasise that these models rely on fundamentally similar principles. In particular, the models include both feedforward and feedback connections, thereby allowing information about the errors made by the network to propagate throughout the network without requiring an external program to compute the errors. Furthermore, these dynamics, as well as the synaptic plasticity, can be described within a common framework of energy minimisation. We divide the reviewed models in two classes differing in how the errors are represented, and we summarise them in the following sections.

### Temporal-Error Models

This class of model encodes errors in the differences in neural activity across time. The first model in this class is the contrastive learning model [37]. It relies on an observation that weight changes proportional to an error (difference between predicted and target pattern) can be decomposed into two separate updates: one update based on activity without the target present and the other update with the target pattern provided to the output neurons [38] (Box 2). Thus, the error back-propagation algorithm can be approximated in a network in which the weights are modified twice: during prediction according to **anti-Hebbian plasticity** and then according to **Hebbian plasticity** once the target is provided and the network converges to an equilibrium (after the target activity has propagated to earlier layers via feedback connections) [37]. The role of the first modification is to ‘unlearn’ the existing association between input and prediction, while the role of the second modification is to learn the new association between input and target.

Box 2 **Temporal-Error Models**

Temporal-error models describe learning in networks with recurrent feedback connections to the hidden nodes (Figure IA). The rate of change of activity of a given node is proportional to the summed inputs from adjacent layers, along with a decay term proportional to the current level of activity (Figure IB). As the network is now recurrent, it is no longer possible to write a simple equation describing how the activity depends on other nodes (such as **Equation 1.1** in Box 1); instead, the dynamics of neurons is described by the differential **Equation 2.1** [72], where ${\dot{x}}_{l}$ denotes the rate of change over time of ***x***_***l***_ (all equations in this figure ignore nonlinearities for brevity).

In the contrastive learning model, the weight modifications based on errors are decomposed into two separate changes occurring at different times. To understand learning in this model, it is easiest to consider how the weights connecting to the output layer are modified. Substituting **Equation 1.4** into **Equation 1.3**, we see in **Equation 2.2** that the weight modification required by the back-propagation algorithm can be decomposed into two terms. The first term corresponds to anti-Hebbian plasticity that should take place when the output activity is predicted based on the input propagated through the network. The second term corresponds to Hebbian plasticity that should take place when the output layer is set to the target pattern. O’Reilly [37] demonstrated that in the presence of backward connections, the information about the target pattern propagates to earlier layers, and an analogous sequence of weight modifications in the hidden layers also approximates a version of the back-propagation algorithm for recurrent networks [72].

In the continuous update model, the output nodes are gradually changed from the predicted pattern (*x*_3_|_¬*t*_) towards the target values (*t*), as shown for a sample neuron in Figure ID. Thus, the temporal derivative of output activity (${\dot{x}}_{3}$) is proportional to (*t* −* x*_3_|_¬*t*_), that is, to the error on the output (defined in **Equation 1.4**). Hence, the weight modification required by back-propagation is simply equal to the product of presynaptic activity and the rate of change of the postsynaptic activity (**Equation 2.3**).

Alt-text: Box 2

**Figure I:**
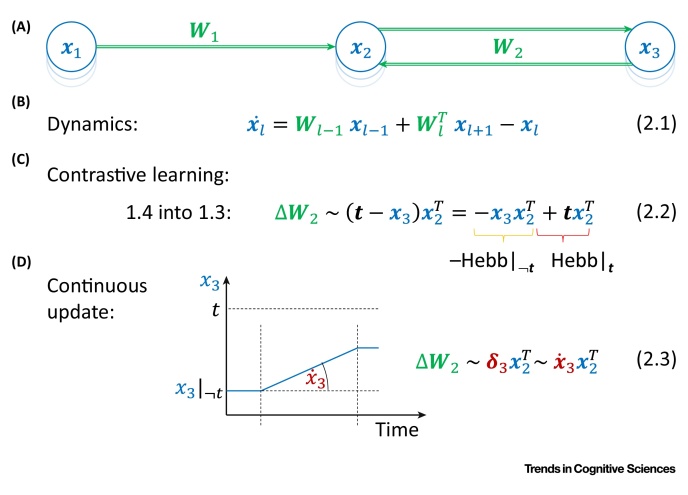
Temporal-Error Models. (A) Network architecture. (B) Dynamics. (C) Contrastive learning. (D) Continuous update.

Although the weight modifications in the contrastive learning model involve locally available information, implementing them biologically would require a global signal informing the network which phase it is in (whether the target pattern influences the network or not) as that determines whether the plasticity should be Hebbian or anti-Hebbian. It is not clear whether such a control signal exists in the brain. This concern can be alleviated if the determination of learning phases is coordinated by information locally available in the **oscillatory rhythms** [39], such as hippocampal theta oscillations [40]. In these models, the neurons in the output layer are driven by feedforward inputs in one part of the cycle and forced to take the value of the target pattern in the other.

The complications of separate phases have been recently addressed in the continuous update model [11], where during training the output neuron activities are gradually changed from the predicted pattern towards the target. In this case, the rate of change of the output units is proportional to the error terms (Box 2). Consequently, the weight modification required by the back-propagation algorithm could arise from local plasticity based on the rate of change of activity. Although the continuous update model does not involve two different learning rules during prediction and learning, it still requires a control signal indicating whether the target pattern is present or not, because plasticity should not take place during prediction.

### Explicit-Error Models

In this section, we describe alternative models that do not require control signals but as a trade-off have more complex architectures that explicitly compute and represent errors.

It has been recently noticed 14, 41 that the error back-propagation algorithm can be approximated in a widely used model of information processing in hierarchical cortical circuits called predictive coding [42]. In its original formulation, the predictive coding model was developed for **unsupervised learning**, and it has been shown that when the model is presented with natural images, it learns representations similar to those in visual cortex [42]. Predictive coding models have also been proposed as a general framework for describing different types of information processing in the brain [43]. It has been recently shown that when a predictive coding network is used for **supervised learning**, it closely approximates the error back-propagation algorithm [14].

An architecture of a predictive coding network contains **error nodes** that are each associated with corresponding **value nodes**. During prediction, when the network is presented with an input pattern, activity is propagated between the value nodes via the error nodes. The network converges to an equilibrium, in which the error nodes decay to zero and all value nodes converge to the same values as the corresponding artificial neural network (Box 3). During learning, both the input and the output layers are set to the training patterns. The error nodes can no longer decrease their activity to zero; instead, they converge to values as if the errors had been back-propagated [14]. Once the state of the predictive coding network converges to equilibrium, the weights are modified, according to a Hebbian plasticity rule. These weight changes closely approximate that of the back-propagation algorithm.

Box 3 **Predictive Coding Model**

Predictive coding networks include error nodes each associated with corresponding value nodes (Figure IA). The error nodes receive inhibition from the previous layer and excitation from the corresponding value nodes and thus compute the difference between them (**Equation 3.1**). The value nodes get feedforward inhibition from corresponding error nodes and feedback from the error nodes in the next layer. In the predictive coding network, the value nodes act as integrators, so they add their input to their current activity level (**Equation 3.2**).

During prediction, when the network is presented only with an input pattern, the information is propagated between the value nodes via the error nodes. As the output layer is unconstrained, the activity of error nodes converges to zero, because the value nodes change their activity until the feedback they send to their corresponding error nodes balances the feedforward inhibition received by error nodes. At this state, the left side of **Equation 3.1** is equal to 0, and by rearranging terms (Figure IC), we observe that the activity of value nodes is equal to the weighted sum of value nodes in the previous layer, exactly as in artificial neural networks [**Equation 1.1** with f(x)=x].

During learning, when the network is presented with both input and target patterns, the activity of error nodes may not decrease to zero. Learning takes place when the network is in equilibrium (${\dot{x}}_{l}=0$). At this stage the left side of **Equation 3.2** is equal to 0, and by rearranging terms (Figure ID), we observe that the activity of error nodes is equal to a weighted sum of errors from the layer above, bearing the same relationship as in the back-propagation algorithm [**Equation 1.5** with f(x)=x]. At convergence, the weights are modified according to **Equation 1.3**, which here corresponds to Hebbian plasticity dependent on the activity of pre- and postsynaptic neurons.

Alt-text: Box 3

**Figure I:**
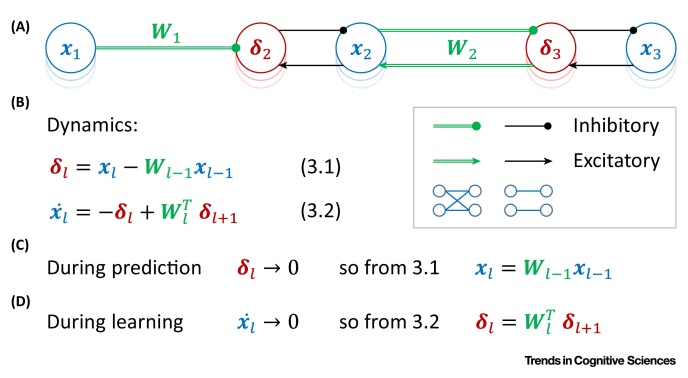
Predictive Coding. (A) Network architecture. Blue and red circles denote the value and error nodes, respectively. Arrows and lines ending with circles denote excitatory and inhibitory connections, respectively; green double lines indicate connections between all neurons in one layer and all neurons in the next layer, while single black lines indicate within layer connections between a corresponding error and value node (see key). (B) Dynamics (for a simple case of linear function *f*; for details of how nonlinearities can be introduced, see [14]). (C) Prediction. (D) Learning.

An important property of the predictive coding networks is that they work autonomously: irrespective of the target pattern being provided, the same rules for node dynamics and plasticity are used. If the output nodes are unconstrained, the error nodes converge to zero, so the Hebbian weight change is equal to zero. Thus, the networks operate without any need for external control except for providing different inputs and outputs. However, the one-to-one connectivity of error nodes to their corresponding value nodes is inconsistent with diffused patterns of neuronal connectivity in the cortex.

A solution to this inconsistency has been proposed in several models in which the error is represented in dendrites of the corresponding neuron 44, 45, 46. In this review article, we focus on a popular model called the dendritic error model [13]. This model describes networks of pyramidal neurons and assumes that the errors in the activity of pyramidal neurons are computed in their **apical dendrites**. In this model, the apical dendrites compare the feedback from the higher levels with a locally generated prediction of higher-level activity computed via interneurons.

An easy way to understand why such an architecture approximates the back-propagation algorithm is to notice that it is closely related to predictive coding networks, which approximate artificial neural networks. Simply rearranging the equations describing the dynamics of predictive coding model gives a description of a network with the same architecture as the dendritic error model, in which dendrites encode the error terms (Box 4).

Box 4 **Dendritic Error Model**

The architecture of the dendritic error model [13] is shown in Figure IA. In this network, the activity is propagated through the layers via connections between pyramidal neurons. The errors in the activity of pyramidal neurons are computed in their apical dendrites.

The relationship between predictive coding and dendritic error models can be established by observing that substituting the definition of error nodes from the predictive coding model, **Equation 3.1**, into **Equation 3.2**, produces **Equation 4.1**, which describes the dynamics of pyramidal neurons in Figure IA. The right side of **Equation 4.1** consists of four terms corresponding to various connections in the figure. The first is simply a decay, the second is a feedforward input from the previous layer, the third is a feedback from the layer above, and the fourth term is a within layer recurrent input. This last term has a negative sign, while pyramidal neurons are excitatory, so it needs to be provided by interneurons. If we assume that the interneurons have activity ***i***_*l*_ =*** W***_*l*_***x***_*l*_, they need to be connected with the pyramidal neurons via weights ***W***_*l*_.

The key property of this network is that when it converges to the equilibrium, the neurons with activity ***x***_*l*_ encode their corresponding error terms ***δ***_*l*_ in their apical dendrites. To see why this is the case, note that the first two terms on the right of **Equation 4.1** are equal to −***δ***_*l*_ according to the definition of **Equation 3.1**. At equilibrium ${\dot{x}}_{l}=0$, the two last terms in **Equation 4.1** must be equal to ***δ***_*l*_ (so that the right-hand side of **Equation 4.1** adds up to 0), and it is these two terms that define the input to the apical dendrite. As the errors ***δ***_*l*_ are encoded in apical dendrites, the weight modification required by the back-propagation algorithm (**Equation 1.3**) only involves quantities encoded in pre- and postsynaptic neurons.

Appropriately updating weights between pyramidal and interneurons is more challenging. This is because the interneurons must learn to produce activity encoding the same information as the higher-level pyramidal neurons. To allow training of the interneurons, the dendritic error model includes special one-to-one connections to the interneurons from corresponding higher-level pyramidal neurons (black dashed arrows in Figure IA).

Alt-text: Box 4

**Figure I:**
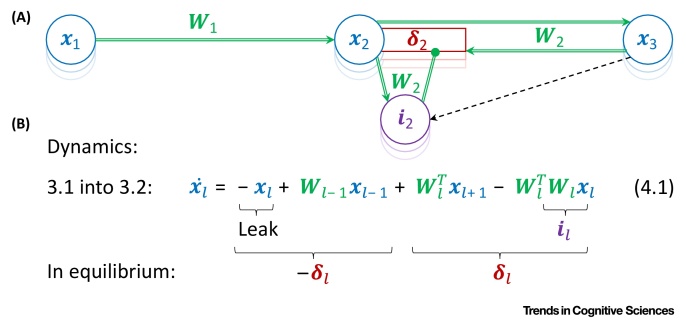
Dendritic Error Model. (A) Network architecture. Blue circles indicate pyramidal neurons, red rectangles indicate their apical dendrites, and purple circles denote interneurons. (B) Dynamics.

As the error term is now encoded within a neuron’s compartment, the update of weights between pyramidal neurons required by the back-propagation algorithm corresponds to local synaptic plasticity. Error information can be transmitted from the apical dendrite to the rest of the neuron through internal signals. For example, a recent computational model proposed that errors encoded in apical dendrites can determine the plasticity in the whole neuron [12]. The model is based on observations that activating apical dendrites induces **plateau potentials** via calcium influx, leading to a burst of spikes by the neuron [47]. Such bursts of spikes may subsequently trigger synaptic plasticity 48, 49.

Although the dendritic error network makes significant steps to increase the biological realism of predictive coding models, it also introduces extra one-to-one connections (dotted arrow in Box 4) that enforce the interneurons to take on similar values to the neurons in next layer and thus help them to predict the feedback from the next level. Furthermore, the exact dynamics in the dendritic error model are much more complex than that given in Box 4, as it describes details of changes in membrane potential in multiple compartments. Nevertheless, it is important to highlight that the architecture of dendritic error networks can approximate the back-propagation algorithm, and it offers an alternative hypothesis on how the computations assumed by the predictive coding model could be implemented in cortical circuits.

## Comparing the Models

Given the biological plausibility of the above-mentioned models, in this and the coming sections, we compare the models in terms of their computational properties (as more efficient networks may be favoured by evolution) and their relationships to experimental data (summarised in Table 1).

**Table 1:** Comparison of Models

		Temporal-error model		Explicit-error model	
		Contrastive learning	Continuous update	Predictive coding	Dendritic error
Properties^a^	Control signal	Required	Required	Not required	Not required
	Connectivity	Unconstrained	Unconstrained	Constrained	Constrained
	Propagation time	L-1	L-1	2L-1	L-1
	Pre-training	Not required	Not required	Not required	Required
Error encoded in		Difference in activity between separate phases	Rate of change of activity	Activity of specialised neurons	Apical dendrites of pyramidal neurons
Data accounted for		Neural responses and behaviour in a variety of tasks	Typical spike-time-dependent plasticity	Increased neural activity to unpredicted stimuli	Properties of pyramidal neurons
MNIST performance^b^		∼2–3	–	∼1.7	∼1.96

a Green indicates properties desired for biological plausibility, while red indicates less desired properties.

b These are error percentages reported on a testing set in a benchmark task of handwritten digit classification (lower is better), for predictive coding [14], dendritic error [13], and contrastive learning models [22] (in this simulation, the output neurons were not set to the target pattern, but slightly moved or ‘nudged’ towards it). We are not aware of reported simulations of the continuous update model on this benchmark problem. MNIST, Modified National Institute of Standards and Technology database.

### Computational Properties

For correct weight modification, the temporal-error models require a mechanism informing whether the target pattern constrains the output neurons, while the explicit-error models do not. However, as a trade-off, the temporal-error models have simpler architectures, while the explicit-error models need to have intricate architectures with certain constraints on connectivity, and both predictive coding and the dendritic error model include one-to-one connections in their network structure. As mentioned, there is no evidence for such one-to-one connectivity in the neocortex.

The models differ in the time required for signals to propagate through the layers. To make a prediction in networks with *L* layers, predictive coding networks need to propagate information through 2*L* − 1 synapses, whereas the other models only need to propagate through *L* − 1 synapses. This is because in a predictive coding network, to propagate from one layer to the next, the information must travel via an error neuron, whereas in the other models the information is propagated directly to the neurons in the layer above. There is a clear evolutionary benefit to propagating information via fewer synapses, as it would result in faster responses and a smaller number of noise sources.

In the dendritic error model, for errors to be computed in the dendrites, the inhibitory interneurons first need to learn to predict the feedback from the higher level. Thus, before the network can learn feedforward connections, ideally the inhibitory neurons need to first be pre-trained. Although it has been shown that the feedforward and inhibitory weights can be learned in parallel, learning in the dendritic error model may well be slower as the reported number of iterations required to learn a benchmark task was higher for the dendritic error model [13] than for contrastive learning [22] and predictive coding [14] models. Such statements, however, should be taken with reservations as not only were simulations not necessarily comparable but also computations in standard von-Neumann computers may not be representative of computations in biological hardware.

### Relationship to Experimental Data

The models differ in their predictions on whether errors should be explicitly represented in neural activity. In particular, the predictive coding model includes dedicated neurons encoding errors, and the dendritic error model suggests that errors computed in dendrites may trigger bursts of firing of pyramidal neurons, while in temporal models there is no direct association between error and the overall activity level at a given time. In line with the explicit-error models, increased neural activity has been observed when sensory input does not match the expectations encoded by higher-level areas. For example, responses of neurons in the primary visual cortex were increased at brief intervals in which visual input did not match expectation based on animal movements [50]. An increase in neural activity when expectations about stimuli were violated has also been found with fMRI [51]. Further details are discussed in several excellent reviews 52, 53, 54, 55. The two explicit models differ in predictions on whether errors and values are represented by separate neuronal populations or within the same neurons. Experimental data relevant to this question have been reviewed in an excellent chapter by Kok and de Lange [56]. Although they conclude that there is ‘no direct unequivocal evidence for the existence of separate populations’, they discuss several studies suggesting preferential encoding of errors and values by different neurons. For example, in a part of visual cortex (inferior temporal cortex), the inhibitory neurons tended to have higher responses to novel stimuli, while excitatory neurons typically produced highest response for their preferred familiar stimuli [57]. Kok and de Lange point that these responses may potentially reflect error and value nodes, respectively [56].

**Figure 1:**
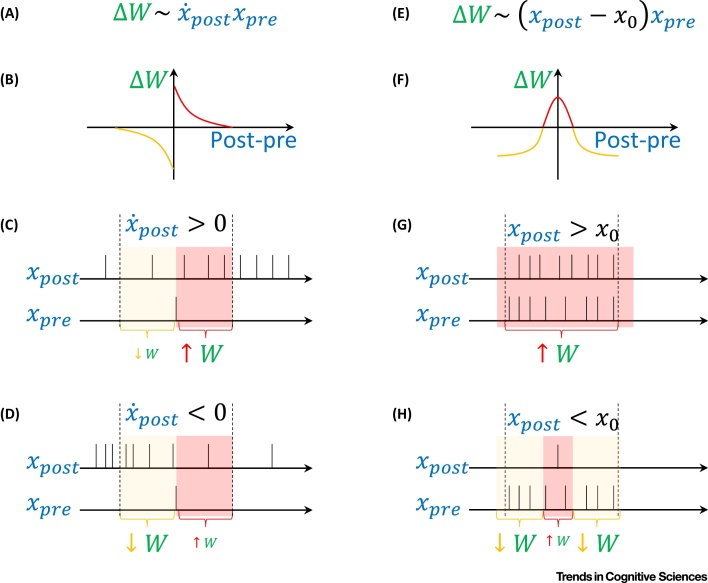
Relationship between Learning Rules and Spike-Time-Dependent Plasticity. (A) Plasticity dependent on the rate of change of postsynaptic activity, illustrated by the left column of panels. (B) Asymmetric spike-time-dependent plasticity often observed in cortical neurons [59]. The curve schematically shows the change in synaptic weights as a function of the difference between the timings of postsynaptic and presynaptic spikes. Red and orange parts of the curve correspond to increases and decreases in synaptic weights, respectively. (C) Strengthening of a synaptic weight due to increasing postsynaptic activity. Hypothetical spike trains of two neurons are shown. The top sequence corresponds to an output neuron, which increases its activity over time towards the target (see Figure ID in Box 2). The bottom sequence corresponds to a neuron in the hidden layer; for simplicity, only a single spike is shown. The pink and yellow areas correspond to spike timings in which the weights are increased and decreased, respectively. In these areas the differences in spike timing result in weight changes indicated by red and orange parts of the curve in the panel B. (D) Weakening of weight due to decrease in postsynaptic activity. (E) Plasticity dependent on postsynaptic activity, illustrated by the right column of panels. In the equation, *x*_0_ denotes the baseline firing rate. (F) Symmetric spike-time-dependent plasticity, where weight change depends on spike proximity. (G) Increase in synaptic weight due to high activity of the postsynaptic neuron. (H) Decrease in synaptic weight when the postsynaptic neurons is less active.

Each model accounts for specific aspects of experimental data. The models based on contrastive learning rules have been shown to reproduce neural activity and behaviour in a wide range of tasks [58]. The learning rule in the continuous update model (in which the synaptic modification depends on the rate of change of the postsynaptic neuron; Figure 1A), can be implemented with classic spike-time-dependent plasticity (Figure 1B) [11]. In this form of plasticity, the direction of modification (increase or decrease) depends on whether the spike of a presynaptic neuron precedes or follows the postsynaptic spike [59]. Figure 1C shows the effect of such plasticity in a case when the postsynaptic neuron increases its firing. If the postsynaptic spike follows the presynaptic spike, the synaptic weight is increased (pink area), while if the postsynaptic spike precedes the presynaptic spike, the weight is decreased (yellow area). If the postsynaptic neuron increases its firing rate (as in the example), there will be more postsynaptic spikes in pink than in yellow area on average, so the overall weight change will be positive. Analogously, the weight is weakened if the postsynaptic activity decreases (Figure 1D). In summary, with asymmetric spike-time-dependent plasticity, the direction of weight change depends on the gradient of a postsynaptic neuron activity around a presynaptic spike, as in the continuous update model.

The relationship of spike-time-dependent plasticity to other models requires further clarifying work. Nevertheless, Vogels and colleagues [60] demonstrated that a learning rule in which the direction of modification depends on activity of neurons in equilibrium (Figure 1E), as in the predictive coding model, can arise from an alternate form of spike-time-dependent plasticity. They considered a form of plasticity where the weight is increased by nearly coincident pre- and postsynaptic spikes, irrespectively of their order, and additionally the weight is slightly decreased by each presynaptic spike. The overall direction of weight modification in this rule is shown in Figure 1F. Such a form of plasticity may exist in a several types of synapse in the brain [61]. Figure 1G illustrates that with such plasticity, the weights are increased if the intervals between pre- and postsynaptic spikes are short, which is likely to occur when the two neurons have high activity. When the postsynaptic neuron is less active (Figure 1H), the short intervals (pink area) are less common, while longer intervals are more common (yellow area), so overall the weight change is negative. In summary, with symmetric spike-time-dependent plasticity the direction of weight change depends on whether the postsynaptic neuron activity is above or below a certain level (which may correspond to a baseline level typically denoted with zero in computational models), as in the predictive coding model.

The dendritic error model describes the computations in apical dendrites of pyramidal neurons and features of cortical micro-circuitry such as connectivity of a group of interneurons called the **Martinotti cells**, which receive input from pyramidal neurons in the same cortical area [62] and project to their apical dendrites [63]. Furthermore, there is some evidence that inhibitory interneurons also receive feedback from higher areas in the cortical hierarchy [64].

## Integrating Models

The above-mentioned comparison shows that each model has its own computational advantages, accounts for different data, and describes plasticity at different types of synapses. It is important to note that the cortical circuitry is much more complicated than any of the proposed models’ architectures. Therefore, the models presented above need not be viewed as competitors but may be considered as descriptions of learning in different motifs of more complex brain networks.

Different classes of models may be more suited for different tasks faced by brain networks. One task engaging the primary sensory areas is predicting the next value of sensory input from the previous ones. A recent modelling study suggests that primary visual and auditory cortices may use an algorithm similar to back-propagation while learning to predict sensory input [65]. This study demonstrated that the temporal properties of receptive field in these areas are similar to those in artificial neural networks trained to predict the next video or audio frames on the basis of past history in clips of natural scenes [65]. In such sensory prediction tasks, the target (i.e., the next ‘frame’ of sensory input) always arrives, so the temporal-error models may be particularly suited for this task, as there is no need for the control signal indicating target presence.

The explicit-error models are suitable for tasks where the timing of target pattern presentation is more uncertain. Although the predictive coding and dendritic error networks are closely related, they also exhibit a trade-off: the predictive coding networks are slow to propagate information once trained, while the dendritic error networks are slower to train. It is conceivable that cortical networks include elements of predictive coding networks in addition to dendritic error motifs, as the cortical networks include many other interneuron types in addition to the Martinotti cells and have a much richer organisation than either model. Such a combined network could initially rely on predictive coding motifs to support fast learning and, with time, the dendritic error models could take over, allowing faster information processing. Thus, by combining different motifs, brain networks may ‘beat the trade-offs’ and inherit advantages of each model.

Furthermore, predictive coding models may describe information processing in subcortical parts of brain networks that do not include pyramidal cells and thus may not be able to support computations of the dendritic error model. Indeed, it has been recently suggested how the predictive coding model can be mapped on the anatomy of cerebellum [66], and the model may also describe aspects of information processing in basal ganglia, where the dopaminergic neurons are well known to encode reward prediction error in their activity [67].

**Figure 2:**
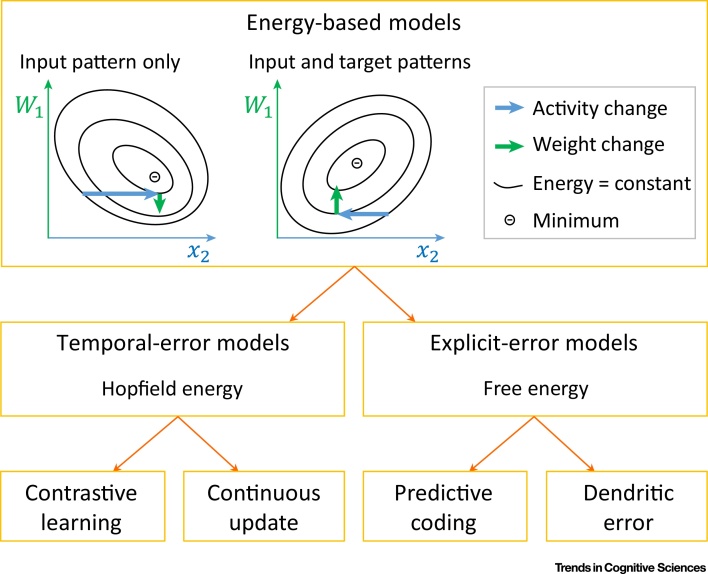
Equilibrium Propagation. The framework considers networks with dynamics described by the minimisation of an energy function. As the activity of these networks converges to an equilibrium, the energy simultaneously decays (blue arrows) to a minimum given the current weights. Once in equilibrium, the weighs are modified (green arrows). It has been shown that network error can be minimised if the synaptic weights are modified in two steps (schematically illustrated by the two displays in the top box; [22]). First, with only the input pattern provided, once the network converges, weights are modified in the direction in which the energy increases. Second, the output layer is additionally constrained to values closer to the target pattern (particular details described in [22]). Constraining the output nodes changes the energy landscape for the units in the middle layers. Once these units converge to a new equilibrium, weights are modified in the direction in which the energy decreases. Scellier and Bengio [22] noted that for temporal-error networks, this procedure gives the contrastive learning rule (**Equation 2.2**). The predictive coding networks, however, converge to an equilibrium in the first step where the free-energy function reaches its global minimum [14]; thus, there is no weight modification required by the equilibrium propagation framework. Therefore, only a single phase (i.e., the second phase) and a single weight update are required in the explicit-error models, and it only involves Hebbian plasticity.

As the brain networks may incorporate elements of different models, it is important to understand how individual models relate to each other and how they can be combined. Such insights have been revealed by a recently proposed framework called **equilibrium propagation** 22, 68. Here, it was noticed that the dynamics of many models of neuronal networks can be defined in terms of the optimisation of a particular function. This function is known as the network energy. For example, recurrently connected networks of excitatory neurons, such as the temporal-error models, under certain assumptions converge to an equilibrium in which strongly connected neurons tend to have similar levels of activity. Indeed, they minimise a function that summarises the dissimilarity in the activity of strongly connected nodes, called the Hopfield energy [69]. The predictive coding networks are also known to minimise a function during their dynamics, called the free energy [70]. The free energy has a particularly nice statistical interpretation, as its negative provides a lower bound on the log probability of predicting the target pattern by the network 70, 71 (in case of supervised learning, this probability is conditioned on the input patterns). Since the dendritic error models have approximately similar dynamics as the predictive coding models, all models reviewed above can be considered as energy-based models described within the equilibrium propagation framework (Figure 2).

The framework also prescribes how synaptic weights should be modified in any network that minimises energy, and the weight modifications in the reviewed models indeed follow this general rule (Figure 2). Importantly, the framework can describe learning in more complex networks, which could include the elements of the different models. For any network for which an energy function can be defined, the framework describes the plasticity rules of individual synapses required for efficient learning.

Nevertheless, the form of energy function minimised by a network may influence its performance. So far, the biologically plausible networks that perform best in a handwritten digit classification task are those that minimise energies analogous to the free energy (Table 1). The superior performance of networks minimising free energy may stem from the probabilistic interpretation of free energy, which ensures that the networks are trained to maximise the probability of predicting target patterns.

## Concluding Remarks

This review article has not been exhaustive of all current biological models but nevertheless has described main classes of recent models; those that represent errors temporally and those that represent them explicitly, as well as a framework unifying these methods. These theoretic results elucidate the constraints required for efficient learning in hierarchical networks. However, much more work needs to be done both empirically and theoretically, for example, on how the networks scale to larger architectures [28], as well as linking theory to neurobiological data (see Outstanding Questions).

It is crucial to map the models implementing efficient deep learning on biological networks in the brain. In particular, mapping the nodes in the model on distinct cell types in the cortex may be a fruitful route to identifying their computational function. The framework of equilibrium propagation (or its future extensions) may prove particularly useful in this endeavour. Based on known patterns of connectivity, models could be defined and their energy function formulated. The framework could then be used to predict properties of synaptic plasticity that could be compared with experimental data, and the results of such comparisons could be iteratively used to improve the models.

Outstanding Questions Are biologically plausible deep learning implementations robust to the lack of symmetry between the feedforward and feedback connections? The four models reviewed use symmetric feedforward and feedback weights. In these models, both sets of weights are modified during learning, and the plasticity rules maintain the symmetry. As mentioned, such symmetry does not exist in brain networks, so it is important to continue investigations into whether biologically plausible networks still perform robustly without weight symmetry.

How can researchers make biologically plausible deep learning implementations scale? Although the above-mentioned models perform well on some tasks, it is unclear whether they scale to larger problems. This is in part due to the multiple iterations required to update node activity via network dynamics. The number of iterations required does not currently scale well for larger networks. Further work optimising this process is required if high depth networks are to be trained.

How can efficient learning of temporal sequences be implemented in biological networks? The models reviewed above focus on a case of static input patterns, but the sensory input received by the brain is typically dynamic, and the brain has to learn to recognise sequences of stimuli (e.g. speech). To describe learning in such tasks, artificial neural networks have been extended to include recurrent connections among hidden units, which provide a memory of the past. It is important to extend the models reviewed above for learning through time.

How can the dynamics of neural circuits be optimised to support efficient learning? This question can be first studied in models of primary sensory areas predicting sensory input from its past values. In such tasks, the dynamics will play an important role, as networks need to generate their predictions at the right time to compare it with incoming sensory data.

## Glossary

**Anti-Hebbian plasticity**: synaptic weight modifications proportional to the negative product of the activity of the pre- and postsynaptic neurons. Thus, if both neurons are highly active, the weight of connection between them is reduced.
**Apical dendrite**: a dendrite emerging from the apex of a pyramidal neuron (i.e., from the part of a cell body closest to the surface of the cortex).
**Artificial neural networks**: computing systems loosely based on brain networks. They consist of layers of ‘neurons’ communicating with each other via connections of different weights. Their task is to transform input patterns to particular target patterns. They are trained to predict target patterns in a process in which weights are modified according to the error back-propagation algorithm.
**Deep learning**: learning in artificial neural networks with more than two layers (often >10). Deep networks have shown much promise in the field of machine learning.
**Equilibrium propagation**: a principled framework for determining network dynamics and synaptic plasticity within energy-based models.
**Error back-propagation**: the main algorithm used to train artificial neural networks. It involves computations of errors associated with individual neurons, which determine weight modifications.
**Error node**: neuron type of predictive coding networks. They compute the difference between a value node and its higher-level prediction.
**Hebbian plasticity**: synaptic weight modifications proportional to the product of the activity of the pre- and postsynaptic neurons. It is called Hebbian in computational neuroscience, as it captures the idea of Donald Hebb that synaptic connections are strengthened between co-active neurons.
**Input pattern**: a vector containing the activity levels to which the neurons in the input layer are set. For example, in the handwritten digit classification problem, an input pattern corresponds to a picture of a digit. Here, the input pattern is a vector created by concatenating rows of pixels in the image, where each entry is equal to the darkness of the corresponding pixel.
**Martinotti cells**: small interneurons found in cortex.
**Oscillatory rhythms**: rhythmic patterns of neural activity, with activity of particular cells oscillating between higher and lower values.
**Plateau potential**: a sustained change in a membrane potential of a neuron, caused by persistent inwards currents.
**Predicted pattern**: a vector of activities generated by the network in the output layer, by propagating the input pattern through layers. In the handwritten digit classification problem, the output layer has ten neurons corresponding to ten possible digits. The activity of each output neuron encodes the network’s prediction for how likely the input pattern is to represent a particular digit.
**Pyramidal neuron**: an excitatory neuron with conically shaped cell body. Found in the cerebral cortex, hippocampus, and amygdala.
**Spike-time-dependent plasticity**: synaptic weight modification that depends on the relative timing between pre- and postsynaptic firing.
**Supervised learning**: a class of tasks considered in machine learning, where both an input and a target pattern are provided. The task for the algorithms is to learn to predict the target patterns from the input patterns.
**Target pattern**: a vector of activity in the output layer, which the network should generate for a given input pattern. For example, in the handwritten digit classification problem, the target pattern is equal to 1 at the position corresponding to the class of the corresponding image and is equal to 0 elsewhere.
**Unsupervised learning**: a class of tasks considered in machine learning where only an input pattern is provided (e.g., an image of a handwritten digit). The task for the learning algorithm is typically to learn an efficient representation of the data.
**Value node**: neuron type of predictive coding networks. Their activity represents the values computed by the network.
